# Bacteriophage Therapy: Discovery, Development, and FDA Approval Pathways

**DOI:** 10.3390/ph18081115

**Published:** 2025-07-26

**Authors:** Sarfaraz K. Niazi

**Affiliations:** College of Pharmacy, University of Illinois, Chicago, IL 60612, USA; sniazi3@uic.edu; Tel.: +1-312-297-0000

**Keywords:** bacteriophage therapy, FDA approval, antimicrobial resistance, antimicrobial, regulatory pathway, clinical trials, GMP manufacturing, international regulation, Soviet phage therapy

## Abstract

The escalating global crisis of antimicrobial resistance, responsible for approximately 1.27 million deaths in 2019, has catalyzed renewed interest in bacteriophage therapy as a viable therapeutic alternative. With projections indicating that drug-resistant bacteria could cause over 39 million deaths worldwide by 2050, developing alternative antimicrobial strategies has become critically urgent. This comprehensive review examines the scientific foundation of bacteriophage therapy, traces its historical development from early Soviet applications through contemporary regulatory frameworks, and provides strategic guidance for developers seeking FDA approval for bacteriophage-based therapeutics. We analyze the current regulatory landscape across major jurisdictions, including manufacturing requirements and clinical development pathways essential for successful market authorization. Approximately 90 clinical trials involving bacteriophages are ongoing worldwide, with 41 studies in the United States demonstrating significant momentum in this field.

## 1. Introduction

The emergence of multidrug-resistant bacterial pathogens represents one of the most pressing challenges in modern medicine, fundamentally threatening the efficacy of conventional antibiotic therapy [1]. The World Health Organization’s 2019 report documented approximately 1.27 million deaths directly attributable to antibiotic-resistant bacteria, with specific bacterial species such as drug-resistant *Pseudomonas aeruginosa* and *Staphylococcus aureus* contributing significantly to this mortality burden [2,3]. Recent projections by international researchers indicate that over the next 25 years, the number of deaths caused by drug-resistant bacteria could exceed 39 million worldwide, with an additional 169 million related deaths, potentially surpassing cancer as a leading cause of mortality [3,4]. This alarming trajectory has prompted the pharmaceutical industry and regulatory agencies to explore innovative therapeutic approaches, with bacteriophage therapy emerging as a promising alternative to traditional antibiotics [5,6].

The resurgence of interest in bacteriophage therapy has been driven not only by urgent medical need but also by advances in our understanding of phage biology, improvements in manufacturing technologies, and evolving regulatory frameworks that accommodate these unique biological therapeutics [7,8]. Unlike broad-spectrum antibiotics that indiscriminately target both pathogenic and commensal bacteria, bacteriophages offer the potential for antimicrobial therapy with minimal disruption to the host microbiome [9]. This specificity, combined with their ability to evolve alongside bacterial targets and their capacity for self-amplification at infection sites, positions bacteriophages as valuable tools in the fight against antimicrobial resistance [10].

The historical development of bacteriophage therapy offers essential lessons for contemporary regulatory strategies, particularly the extensive Soviet experience that maintained institutional knowledge and clinical applications throughout the antibiotic era [11]. Despite being discovered in the early 20th century, bacteriophage therapy was largely abandoned in Western medicine due to several factors: the advent of broad-spectrum antibiotics that were easier to standardize and manufacture, lack of understanding of phage biology and mechanisms of action, inconsistent clinical results due to poor quality control, and the influence of negative reviews in prominent medical journals [12]. However, the urgent need for new antimicrobial solutions has brought phage therapy back to the forefront of medical research [13]. Understanding this historical context, combined with analysis of current regulatory frameworks across major jurisdictions, provides essential guidance for developers seeking to navigate the complex pathway to market authorization [14,15].

## 2. Scientific Nature and Biology of Bacteriophages

### 2.1. Fundamental Characteristics and Classification

Bacteriophages, commonly referred to as phages, are viruses that specifically infect and replicate within bacterial cells, representing the most abundant biological entities on Earth with an estimated 10^31^ particles distributed across virtually every environmental niche where bacteria exist [16]. These obligate intracellular parasites exhibit remarkable diversity in their morphology, genomic organization, and life cycles, yet share the fundamental characteristic of bacterial host specificity, which forms the basis of their therapeutic potential [17]. Phages typically consist of a protein capsid head containing the viral genetic material, which may be either DNA or RNA, and often feature a tail structure equipped with specialized receptor-binding proteins that determine host specificity [17,18].

### 2.2. Differences Between Gram-Positive and Gram-Negative Targeting Phages

The structural differences between Gram-positive and Gram-negative bacteria significantly influence the characteristics and mechanisms of action of their respective bacteriophages [19]. Phages targeting Gram-negative bacteria typically possess more complex tail structures with specialized receptor-binding proteins that recognize lipopolysaccharides (LPS) and outer membrane proteins [20]. These phages often employ contractile tail mechanisms to penetrate the dual membrane system characteristic of Gram-negative bacteria [21].

In contrast, phages targeting Gram-positive bacteria generally have simpler tail structures adapted to the thick peptidoglycan cell wall environment [22]. They typically recognize teichoic acids and surface proteins as receptors. The absence of an outer membrane in Gram-positive bacteria allows for various penetration mechanisms, often involving the enzymatic degradation of the peptidoglycan layer [23]. Gram-positive targeting phages frequently produce endolysins with varying substrate specificities compared to those targeting Gram-negative bacteria, reflecting the distinct cell wall compositions of their respective hosts [24,25] (Table 1).

The classification of bacteriophages is primarily based on their life cycle strategies, which can be broadly categorized into lytic and lysogenic cycles [35]. Lytic phages, which are of primary interest for therapeutic applications, undergo a replication cycle that culminates in bacterial cell lysis and the release of progeny phage particles, typically within 20–40 min of infection, depending on the specific phage–host system [28,35]. This rapid multiplication and bacterial destruction mechanism provides the theoretical foundation for phage therapy, as a single infectious phage particle can potentially generate hundreds of offspring while simultaneously eliminating the target pathogen. Lysogenic phages, in contrast, integrate their genetic material into the host bacterial chromosome, remaining dormant until specific environmental triggers induce the lytic cycle [36].

### 2.3. Mechanisms of Bacterial Killing and Specificity

The therapeutic efficacy of bacteriophages derives from multiple mechanisms of bacterial killing that extend beyond simple cell lysis [28,37]. The primary mechanism involves the production of lytic enzymes, including endolysins and holins, which disrupt bacterial cell wall integrity and cause osmotic lysis [24,25]. Recent research has demonstrated that some phages encode additional antimicrobial proteins that can sensitize bacteria to antibiotic treatment or disrupt bacterial biofilm formation, suggesting potential synergistic effects when combined with conventional antimicrobials [38,39].

The exquisite specificity of bacteriophages for their bacterial hosts represents both a significant advantage and a potential limitation for therapeutic applications [27]. This specificity is mediated by the interaction between phage tail fibers or tail spikes and specific receptor molecules on the bacterial cell surface, creating a lock-and-key mechanism that determines host range [22,23]. While this specificity minimizes off-target effects on commensal bacteria, it also necessitates precise pathogen identification, potentially limiting the breadth of coverage that can be achieved with a single phage preparation [26]. Consequently, many therapeutic applications employ phage cocktails containing multiple phages with complementary host ranges to ensure adequate coverage of target pathogens [40,41].

### 2.4. Phage–Bacteria Evolutionary Dynamics

The co-evolutionary relationship between bacteriophages and their bacterial hosts represents a critical consideration for therapeutic development, as it drives both the development of bacterial resistance mechanisms and the corresponding phage adaptations that overcome such resistance [29,42]. Bacteria can develop resistance to phage infection through multiple mechanisms, including modification or loss of surface receptors, development of restriction-modification systems, CRISPR-Cas adaptive immunity, and prophage-mediated immunity [43,44]. However, the evolutionary pressure exerted by phages often comes at a fitness cost to bacteria, potentially reducing virulence or antibiotic resistance in resistant mutants [45,46].

This evolutionary arms race provides opportunities for therapeutic exploitation, as phage resistance may restore antibiotic sensitivity or reduce bacterial pathogenicity [47,48]. Furthermore, the rapid evolution of phages enables the development of adapted variants that can overcome bacterial resistance mechanisms, providing a renewable source of therapeutic agents [9,49]. Understanding these evolutionary dynamics is crucial for designing effective and sustainable phage therapy protocols and predicting long-term treatment outcomes [50,51].

## 3. Historical Perspective and Evolution of Phage Therapy

### 3.1. Early Discovery and Pioneer Era (1915–1940)

The formal discovery of bacteriophages can be traced to the independent observations of Frederick Twort in 1915 and Felix d’Herelle in 1917, who characterized their ability to cause “transmissible bacterial lysis” [52,53]. D’Herelle, who coined the term “bacteriophage,” recognized the therapeutic potential of these agents and initiated the first documented phage therapy in 1919 in Paris, treating patients with bacterial dysentery [53]. This pioneering work established the conceptual foundation for phage therapy and sparked international interest in developing these agents as antimicrobial treatments.

The early decades of phage therapy development were marked by the widespread clinical application of phages across diverse medical conditions, including typhoid fever, cholera, and various wound infections [11]. D’Herelle’s collaboration with physicians worldwide led to the establishment of phage therapy programs in multiple countries, with extensive development in the former Soviet Union under the leadership of George Eliava [54]. During this period, pharmaceutical companies such as Eli Lilly began commercial production of phage preparations, making these treatments widely available to physicians.

### 3.2. Decline and Overshadowing by Antibiotics (1940–1980)

The widespread adoption of penicillin and subsequent antibiotics during the 1940s marked the beginning of a rapid decline in Western interest in phage therapy [30]. Several factors contributed to this transition, including the broad-spectrum activity of antibiotics, which contrasted with the narrow specificity of phages, the improved understanding of antibiotic mechanisms of action, and the challenges associated with standardizing phage production and quality control [55]. An influential, unfavorable review published in the *Journal of the American Medical Association* in the early 1930s may have also contributed to skepticism regarding the efficacy of phage therapy [12].

The advent of the “golden era” of antibiotic discovery, spanning the 1940s to the 1960s, provided physicians with reliable, standardized antimicrobial agents that could be produced at scale with consistent potency and broad-spectrum activity [30]. This period saw the identification of major antibiotic classes, including streptomycin, chloramphenicol, tetracycline, and erythromycin, establishing antibiotics as the dominant paradigm for treating bacterial infections. Consequently, phage therapy was largely abandoned in Western medicine. However, it continued to be developed and used in Eastern European countries, notably Georgia and Poland, where extensive phage therapy programs persisted throughout the era of antibiotics [56].

### 3.3. Soviet Development and Military Applications (1930–1990)

During the Soviet era, bacteriophage therapy underwent extensive development and systematic implementation across military and civilian healthcare systems [57]. The collaboration between Felix d’Herelle and Georgian scientist Giorgi Eliava proved instrumental in establishing the Soviet Union as the global center for phage therapy research and application. Eliava played a central role in developing and promoting the therapeutic uses of bacteriophages in the Union of Soviet Socialist Republics and beyond [11]. It was mainly due to his efforts—and the institute he established—that phage therapy survived in Soviet Georgia during the Cold War [11].

The establishment of the Tbilisi Institute of Microbiology, Epidemiology, and Bacteriophage (now known as the Eliava Institute) represented a significant institutional commitment to the development of phage therapy [54]. D’Herelle intended to move to Tbilisi permanently and live in the French cottage specifically built near the institute, which would be shared by his family and Eliava [54]. While in Georgia, d’Herelle completed his survey work, “Bacteriophage and the Phenomenon of Recovery,” which was translated into Russian by Eliava and published by Tbilisi University Press in 1935 [58].

The political climate of the Soviet Union tragically impacted the development of phage therapy when Eliava was arrested in his house on 22 January 1937 on the accusation of anti-Soviet activity, one of the early victims in the year of the Great Terror [59]. Because of his progressive thinking, tireless activities, and close collaborations with many foreign scientists, including d’Herelle, Eliava became a victim of Stalin’s regime in 1937, declared an “Enemy of the People”, and executed [59]. In Soviet Ukraine, both Mel’nyk and Ruchko were executed, with similar accusations of nationalism and sabotage [60].

Despite these setbacks, the military needs of the Soviet Union pushed phage therapy to the forefront of applied microbiology [13]. As the USSR invaded Finland in 1939–1940 and then joined the allies after Nazi Germany invaded in June 1941, medical trials of bacteriophages expanded [13]. During World War II, the Soviet Union utilized bacteriophages to treat soldiers infected with various bacterial diseases, including dysentery and gangrene [61]. The old Soviet literature indicates that phage therapy was used extensively to treat a wide range of bacterial infections in various specialties, including dermatology, ophthalmology, urology, stomatology, pediatrics, otolaryngology, and surgery [56].

At its height after World War II, the Eliava Institute employed 1300 people [62]. Soviet researchers continued to develop and refine their treatments, publishing their research and results. However, due to the scientific barriers of the Cold War, this knowledge was not disseminated and remained largely confined to the local level [63]. Phage preparations were used for diagnostic, therapeutic, and prophylactic purposes to combat various infectious bacterial diseases.

### 3.4. Resurgence in the Antibiotic Resistance Era (1990-Present)

The resurgence of interest in phage therapy began in the 1990s as the limitations of antibiotic therapy became increasingly apparent through the emergence of multidrug-resistant pathogens [30]. The end of the “golden era” of antibiotic discovery in the 1960s, combined with the reduced financial incentives for antibiotic development due to poor return on investment, created a growing gap between bacterial resistance and therapeutic options [30]. By 2014, only four multinational pharmaceutical companies maintained antibiotic divisions, making the need for alternative antimicrobial strategies critically urgent [55].

The modern renaissance of phage therapy was catalyzed by several high-profile clinical cases that demonstrated the life-saving potential of these treatments [64]. The landmark case of Tom Patterson in 2016 provided a dramatic illustration of phage therapy’s potential, as researchers from the Center for Phage Technology at Texas A&M University and scientists from the U.S. Navy successfully treated a patient with an antibiotic-resistant *Acinetobacter baumannii* infection using personalized phage therapy [64]. This case, widely publicized through media coverage, the patient’s wife, and infectious disease epidemiologist Steffanie Strathdee, helped reignite interest in bacteriophage therapy throughout the medical community and regulatory agencies.

### 3.5. Contemporary Development and Clinical Translation in the Former Soviet States

The production and use of phages for therapy and prophylaxis at the Eliava Institute of Bacteriophage never ceased; however, the scale is significantly smaller than it was before the dissolution of the Soviet Union [65]. The Eliava Institute in Tbilisi, Georgia, remains one of the most active centers for phage therapy worldwide, with phage cocktails commonly sold in pharmacies in Eastern European countries, such as Russia and Georgia [57]. The composition of bacteriophagic cocktails has been periodically modified to add phages effective against emerging pathogenic strains [66].

Dr. Mzia Kutateladze at the Eliava Institute continues the research tradition established by Eliava and d’Herelle, focusing on bacteriophage therapy applications across diverse clinical conditions [67]. The Institute maintains an extensive library and research center that serves as a repository for decades of knowledge and experience in phage therapy [60]. The systematic documentation of clinical cases from the Eliava Institute has provided valuable real-world evidence supporting the safety and efficacy of phage therapy approaches.

Contemporary phage therapy development at the Eliava Institute emphasizes quality control and standardization while preserving the flexibility required for personalized treatment approaches [31]. The Institute actively collaborates with numerous local and foreign universities and research centers, serving as a bridge between the extensive Soviet-era experience and modern regulatory requirements for clinical development [31].

### 3.6. Global Expansion and Contemporary Challenges

The current era of phage therapy development is characterized by significant advances in our understanding of phage biology, improvements in manufacturing technologies, and the establishment of regulatory frameworks specifically designed to accommodate these unique therapeutic agents [18]. Unlike the empirical approaches of the early 20th century, contemporary phage therapy development employs sophisticated molecular techniques for phage characterization, genomic analysis to ensure safety, and rational design approaches to optimize therapeutic efficacy [68].

Modern phage therapy programs have been established at major medical centers worldwide, with notable examples including the Center for Innovative Phage Applications and Therapeutics (IPATH) at the University of California, San Diego, the Tailored Antibacterials and Innovative Laboratories for Phage (TAILΦR) at Baylor College of Medicine, and Phage Australia [69,70]. These programs combine clinical treatment capabilities with research infrastructure to advance the field through systematic documentation of treatment outcomes and the development of standardized protocols for phage therapy implementation.

## 4. Discovery and Identification of Bacteriophages

**Figure 1:**
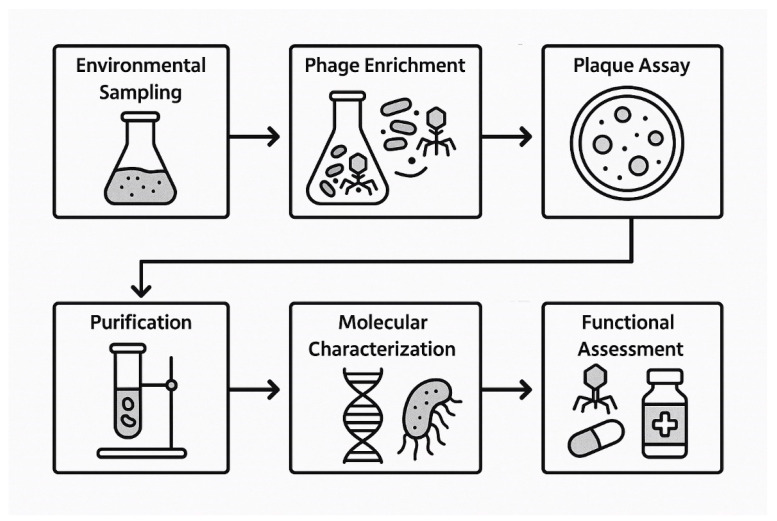
Flowchart of bacteriophage discovery.

Bacteriophage isolation and characterization follow a systematic sequence of experimental steps designed to identify lytic viruses capable of infecting specific bacterial hosts [71,72]. The general workflow includes environmental sampling, phage enrichment, plaque assay, purification, and molecular and functional characterization [71,72] (Figure 1).

### 4.1. Steps of Discovery

Samples are collected from phage-rich environments such as sewage effluent, soil, and river water, as these niches are known to harbor diverse bacteriophage populations [73]. These samples serve as the primary source for isolating phages targeting the selected bacterial host strains.

To selectively amplify phages specific to the target bacteria, the samples are incubated with exponentially growing host bacterial cultures under aerobic conditions at 37 °C for 18–24 h [74]. This enrichment step enhances phage replication by promoting infection and lysis of the host bacteria.

Post-incubation, the cultures are centrifuged and filtered through 0.22 µm membrane filters to remove bacterial cells and debris [75]. The resulting filtrate, containing putative bacteriophages, is used for downstream plaque assays [75].

The soft agar overlay technique is used to detect lytic phages [76]. The filtrate is mixed with the host bacteria and overlaid onto nutrient agar plates. Plaque formation after incubation at 37 °C indicates zones of bacterial lysis, representing sites of individual phage infection [76].

Well-isolated plaques are aseptically picked and suspended in SM buffer [77]. Multiple rounds of plaque picking are performed to ensure the clonality and purity of each phage isolate [77].

The spectrum of bacterial strains susceptible to each isolated phage is determined using spot or drop assays [78]. These assays involve spotting phage suspensions on lawns of various clinical or reference bacterial strains [78].

Phages are visualized via transmission electron microscopy (TEM) following negative staining [79]. This enables classification based on tail structure and capsid morphology [79].

Genomic DNA is extracted from high-titer phage lysates and sequenced using next-generation sequencing [80]. Annotation is performed to identify genes associated with lytic replication and host specificity, and to confirm the absence of undesirable elements such as virulence or antibiotic resistance genes [80].

Genomic and functional assays are used to classify phages as strictly lytic or temperate [36]. Lytic phages are prioritized for therapeutic applications due to their predictable bactericidal activity [36].

Thermal and pH stability assays are performed to determine the robustness of the compound under physiological and storage conditions [81]. Additional tests assess biofilm disruption, synergism with antibiotics, and activity in complex biological matrices such as serum or sputum [81].

### 4.2. Predictive Approaches

Theoretically, it is possible to predict certain structural aspects of a bacteriophage based on its bacterial host, but this remains a complex and probabilistic endeavor rather than a deterministic one [82,83]. Bacteriophages evolve in intimate co-adaptation with their bacterial hosts, particularly through receptor-binding interactions, such that specific host surface structures (e.g., lipopolysaccharides, outer membrane proteins, and flagella) select for phage tail fiber configurations capable of initiating infection [19,21]. In some cases, insights into host receptor composition can allow inferences about the likely phage morphology—for instance, *E. coli* often harbors T-even phages with contractile tails that recognize specific LPS variants [20,84]. Additionally, bacterial CRISPR spacer sequences, which derive from prior phage infections, can be matched to known phage genomes, enabling reverse-engineering of phage proteins, some of which (e.g., capsid or tail fiber proteins) may be structurally modeled using tools such as AlphaFold2 or RosettaFold [85,86,87]. Comparative metagenomic tools, such as PHASTER and VirSorter, can further assist in identifying prophage remnants or phage–host associations, particularly in bacterial genomes that carry integrated or cryptic phage sequences [88,89].

However, the vast structural diversity and mosaicism of phage genomes—where structural modules are frequently recombined across taxa—limit the reliability of direct host-to-structure inference [18,90]. While partial predictions, such as tail morphology class or receptor-binding domain characteristics, may be feasible when supported by sequence data or CRISPR matches, the precise 3D architecture of a phage, its lifecycle classification (lytic vs. temperate), and functional infectivity generally require experimental validation through plaque assays, electron microscopy, and genome annotation [68,91,92]. Thus, while computational and bioinformatic advances continue to expand the scope of theoretical phage discovery, structure prediction from a bacterial target remains a guided approximation rather than a definitive projection [93,94].

## 5. Phage Display-Derived Tools

Although bacteriophages are naturally restricted to infecting bacteria, phage display and phage-derived technologies have extended their utility into eukaryotic systems, especially in oncology [95,96]. Through the strategic selection of ligands, chemical modification, and capsid engineering, phages have become versatile tools for targeting cancer, delivering genes, imaging, and vaccine development [97]. This intersection of synthetic biology and virology illustrates how a prokaryotic virus can be retooled to address complex therapeutic challenges in human medicine [98].

Bacteriophages (phages) are viruses that naturally infect and replicate within bacteria, exploiting bacterial surface receptors and molecular machinery [99]. Their host specificity, however, limits their natural infectivity exclusively to prokaryotic cells [100]. Despite this biological restriction, phages have emerged as powerful biotechnological tools, particularly through the advent of phage display technology, which has been extensively applied to the development of novel diagnostic, therapeutic, and targeting platforms for human diseases, including cancer and other genetically altered eukaryotic cells [101,102].

Phage display technology, pioneered by George P. Smith in the 1980s, involves presenting peptides or proteins on the surface of filamentous phages, such as M13 [103]. The phage genome is engineered to express foreign peptide sequences fused to phage coat proteins (commonly pIII or pVIII), enabling high-throughput screening for ligands that bind to specific cellular targets [104,105].

### 5.1. Peptides

One of the landmark applications of this technique was the identification of peptides that are selectively home to tumor vasculature [106]. Pasqualini and Ruoslahti (1996) [106] used in vivo phage display to isolate peptides that bind to tumor endothelial markers, demonstrating that phages could be used to map the “vascular ZIP codes” of tumors and identify cancer-specific ligands. Subsequent studies have expanded this approach to identify peptides targeting various cancer-associated receptors and tissue-specific markers [107,108].

### 5.2. Nanocarriers

Phage display has been instrumental in identifying ligands that guide nanocarriers or conjugated drugs specifically to cancer cells [109]. For instance, peptides that bind HER2, EGFR, or integrins have been identified and used to direct liposomes or nanoparticles to malignant cells, increasing therapeutic specificity while reducing systemic toxicity [110,111]. These targeted delivery systems have shown enhanced therapeutic indices in preclinical cancer models [112,113].

### 5.3. Engineered Phage

While wild-type phages do not infect eukaryotic cells, engineered phage particles have been developed to deliver genetic payloads, such as siRNA, shRNA, or DNA, into human cells [114,115]. Phages modified with mammalian cell-penetrating peptides and nuclear localization signals have shown potential in preclinical models of gene therapy [116,117]. These engineered systems can bypass the natural host restriction and deliver therapeutic nucleic acids to specific cell types [118].

Phage particles displaying tumor-associated antigens (TAAs) have been used to stimulate immune responses against cancer cells [119,120]. For example, phages expressing the HPV16 E7 peptide have been shown to induce cytotoxic T lymphocyte (CTL) responses in murine models, functioning as adjuvant-free, self-adjuvanting vaccine platforms [121,122]. Clinical trials have demonstrated the safety and immunogenicity of phage-based cancer vaccines [123,124].

### 5.4. Chemical Modification

Beyond genetic manipulation, phage capsids can be chemically modified or emptied of genetic material to serve as nano-carriers [125,126]. These capsids offer excellent biocompatibility, stability, and modifiability [127]. Gold-coated phage particles have been utilized in photothermal therapy, where phages accumulate at tumor sites and are subsequently irradiated with near-infrared light, resulting in local heating and tumor ablation [128,129]. Other chemical modifications include PEGylation for enhanced circulation time and conjugation with fluorescent dyes for imaging applications [130,131].

Such synthetic or semi-synthetic systems retain the geometric precision and multivalency of phage surfaces while functioning independently of replication or infection mechanisms [132,133].

It remains biologically implausible for a native bacteriophage to evolve the capability to infect or replicate within eukaryotic cells [134]. The fundamental differences between bacterial and mammalian cell surfaces, internalization pathways, and intracellular machinery form a robust species barrier [135,136]. However, mimicking phage functionality—notably their modular surface display and encapsulation potential—has unlocked their utility beyond their natural domain [137,138].

## 6. Current Regulatory Framework and FDA Pathway

### 6.1. Regulatory Classification and Oversight Structure

Bacteriophage products intended to treat or prevent disease are classified as biological products and drugs under United States federal law [14]. They are regulated by the Center for Biologics Evaluation and Research (CBER) at the Food and Drug Administration (FDA) [14]. This classification reflects the live, replicating nature of therapeutic phages, aligning them with other biological products, such as vaccines and cell therapies, that require specialized regulatory oversight [33]. The regulatory framework applicable to bacteriophage products generally requires that clinical investigations involving human subjects be conducted under an Investigational New Drug (IND) application, following the same pathway established for conventional pharmaceuticals while accommodating the unique characteristics of these biological agents [139].

**Figure 2:**
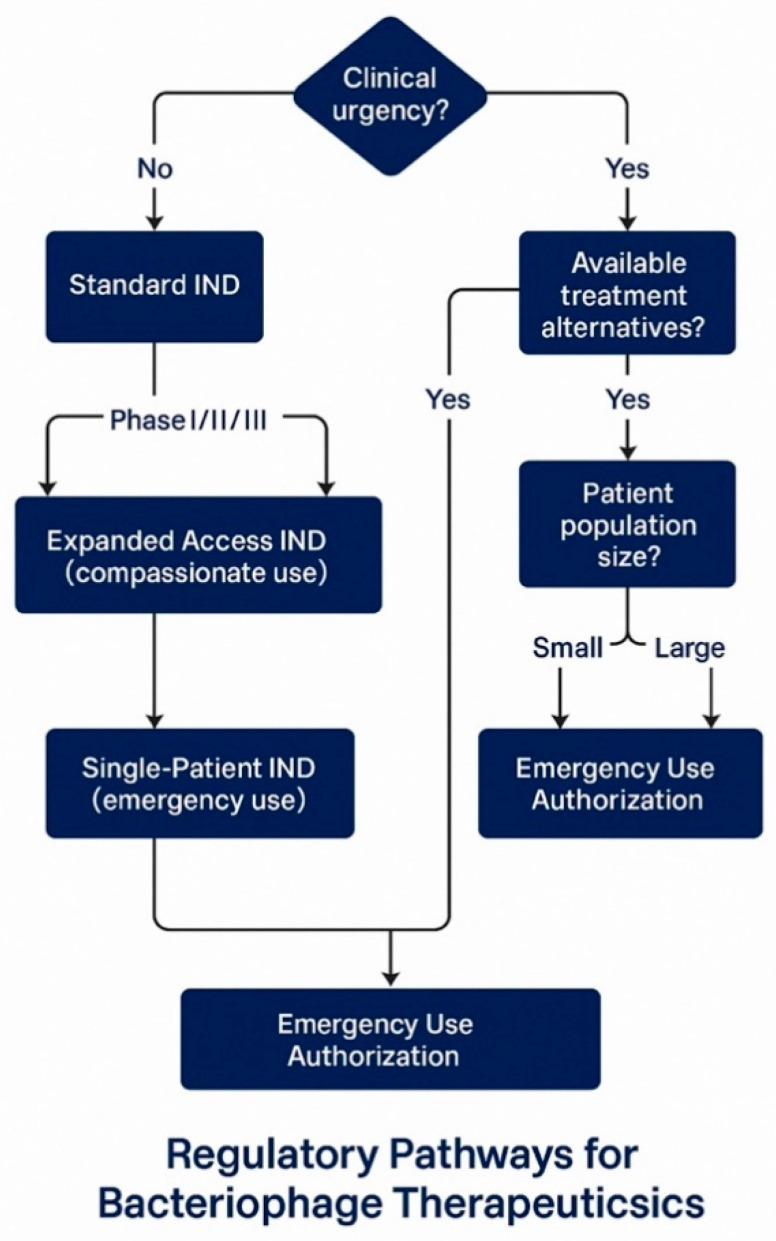
Bacteriophage regulatory pathways.

The FDA’s approach to regulating bacteriophage therapy has evolved significantly since the early 2000s, with the agency demonstrating increasing engagement and support for these innovative treatments [140]. This evolution is exemplified by the FDA’s approval of bacteriophage preparations for food safety applications, beginning with the approval of ListShield™ in 2006 as the first phage-based food additive and continuing with multiple subsequent approvals for agricultural and food safety applications [141]. These precedents have helped establish the regulatory foundation for human therapeutic applications and demonstrate the FDA’s acceptance of bacteriophage technology across multiple application domains [142] (Figure 2, Table 2).

### 6.2. Investigational New Drug Application Pathways

The IND pathway provides several mechanisms for accessing bacteriophage therapy, each designed to address different clinical scenarios and development stages [146]. The standard IND pathway supports systematic clinical development through sequential Phase I, II, and III trials leading to Biologics License Application (BLA) submission for market approval [143]. This pathway requires comprehensive preclinical safety data, detailed manufacturing information, and clinical protocols designed to evaluate safety and efficacy in accordance with established guidelines for anti-infective agents [151].

Expanded access INDs represent a critical pathway for accessing experimental phage therapy in cases where patients have serious or life-threatening conditions, and no comparable treatment options exist [150]. Research at Baylor College of Medicine’s TAILΦR program demonstrates the practical implementation of this pathway, where investigators received FDA approval for compassionate use under an Investigational New Drug application for 12 customized phage therapy cases out of 50 requests [148]. The reasons for the 38 cases that were not adapted included patient improvement in 8 cases, treatment delays exceeding 10 weeks in 8 cases, patient death before treatment initiation in 8 cases, inability to isolate appropriate phages in 5 cases, and unidentified bacteria in 4 cases, illustrating both the potential and limitations of personalized phage therapy approaches [152].

Single-patient Investigational New Drug (IND) applications provide an additional mechanism for emergency access to experimental phage therapy, enabling the treatment of individual patients when standard expanded access protocols are not applicable [149]. This pathway has been successfully utilized in numerous high-profile cases, contributing significantly to our understanding of phage therapy’s safety and efficacy in clinical settings [153]. The cumulative experience with single-patient Investigational New Drug (IND) applications has provided valuable data supporting the broader development of phage therapy and has helped establish clinical protocols that can be scaled to larger patient populations [6].

### 6.3. FDA Engagement and Collaborative Development

The FDA has demonstrated remarkable openness to engaging with the phage therapy community through multiple initiatives designed to facilitate regulatory understanding and streamline development pathways [14]. The agency’s 2021 “Science and Regulation of Bacteriophage Therapy” workshop exemplified this collaborative approach, bringing together researchers from government, academia, and industry to exchange information about regulatory and scientific issues associated with bacteriophage therapy [154]. This three-day virtual workshop featured breakout sessions covering critical topics, including single-patient Investigational New Drug (IND) applications, National Institute of Allergy and Infectious Diseases (NIAID) funding mechanisms, and clinical trial design, providing developers with direct access to regulatory experts and guidance on navigating the approval process.

The FDA’s supportive stance toward phage therapy development is further evidenced by statements from industry leaders who characterize the agency as being “on board” with phage therapy and “very thoughtful and reasonable” in its approach to regulating phage therapeutics [34]. This regulatory environment has enabled the advancement of multiple phage therapy programs through clinical development, with approximately 41 bacteriophage studies currently ongoing in the United States as part of the 90 clinical trials worldwide [155]. The agency’s willingness to engage in pre-IND meetings, provide guidance on clinical trial design, and consider adaptive regulatory pathways reflects a pragmatic approach to addressing the urgent medical need for new antimicrobial treatments.

### 6.4. Regulatory Challenges and Advocacy for Reform

Despite the FDA’s generally supportive approach, significant regulatory challenges remain that may impede the efficient development and approval of bacteriophage therapeutics [156]. Industry experts and patient advocacy groups have identified several areas where regulatory reform could accelerate access to these life-saving treatments while maintaining appropriate safety standards. A prominent advocacy perspective suggests that the FDA should establish a streamlined approval process specifically for phage therapy, recognizing the fundamental differences between phages and traditional drugs [156]. This approach could potentially include adaptive trial designs that facilitate faster iteration and adjustment based on real-world evidence [156].

The current regulatory framework, while adequate for many pharmaceutical products, may be too rigid and costly for phage therapy development, particularly given the time-sensitive nature of many applications and the need for personalized treatment approaches [157]. Recommendations for regulatory reform include relaxing Good Manufacturing Practice standards in the context of producing phages for expanded access to treatment of critically ill patients, which could enable more laboratories to produce phages more quickly and potentially save lives in emergencies. Additionally, enhanced funding and support for research into phage therapy, along with the encouragement of academic and commercial partnerships, could accelerate the development of safe and effective phage treatments.

International harmonization of regulatory standards represents another critical opportunity for improving global access to phage therapy, ensuring that promising treatments developed in other countries can be efficiently evaluated and approved for use in the United States [158]. This harmonization effort would require close collaboration between the FDA and international regulatory bodies to establish mutually acceptable standards for the development, manufacturing, and clinical evaluation of phage therapy.

## 7. Manufacturing and Quality Control Requirements

### 7.1. Good Manufacturing Practice Compliance Framework

The manufacturing of bacteriophage therapeutic products must comply with Good Manufacturing Practice (GMP) standards, which represent the gold standard for ensuring the quality, safety, and efficacy of medicinal products used in clinical investigations and commercial applications [32,159]. The implementation of GMP requirements for phage therapy medicinal products benefits significantly from the extensive regulatory experience with vaccine development, as both product categories involve live biological agents that require specialized manufacturing and quality control approaches [159]. This regulatory precedent provides a foundational framework that can be adapted to address the unique characteristics of bacteriophage products while maintaining the rigorous standards necessary for patient safety.

The GMP compliance framework for bacteriophage products encompasses multiple interconnected elements that must be systematically addressed throughout the manufacturing process [32]. These elements include the establishment of appropriate facilities designed to prevent cross-contamination between different phage products, the implementation of validated manufacturing processes that consistently produce products meeting predetermined specifications, and the development of comprehensive quality control testing programs that verify product identity, purity, potency, and safety [32]. Additionally, GMP compliance requires extensive documentation of all manufacturing activities, training and qualification of personnel involved in production and quality control, and establishment of robust change control systems that ensure product consistency throughout the product lifecycle [159].

### 7.2. Quality by Design Implementation

The implementation of Quality by Design (QbD) principles represents the most effective approach for establishing robust and cost-effective manufacturing processes for bacteriophage drug products [159]. The QbD methodology begins with the definition of a Quality Target Product Profile (QTPP), which specifies the intended clinical setting, route of administration, dosage, container system, and storage requirements for the final drug product [159]. This QTPP subsequently guides the identification of critical quality attributes (CQAs), which represent the biological, chemical, microbiological, and physical characteristics that the product must possess to ensure safety and efficacy.

**Figure 3:**
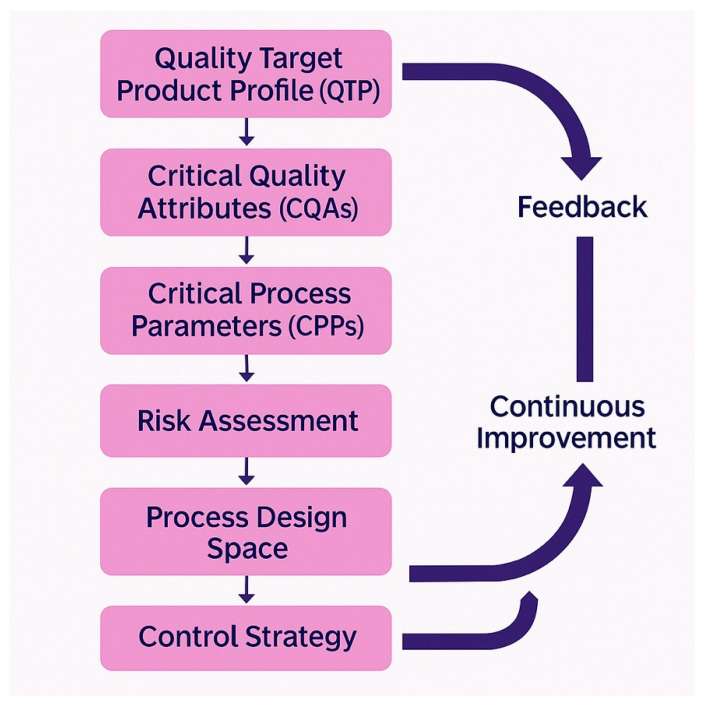
Quality by Design framework for bacteriophage manufacturing.

For bacteriophage-based drug products, the CQAs typically include product identity confirmed through molecular characterization, absence of contaminating phages that could affect product performance or safety, precise titers of each phage present in cocktail formulations, maximum acceptable levels of bacterial endotoxins and other contaminants, appropriate pH for stability and biocompatibility, demonstrated sterility, and defined shelf life under specified storage conditions [8,160]. The systematic identification and control of these critical quality attributes (CQAs) through the Quality by Design (QbD) methodology enables manufacturers to develop robust processes that consistently deliver products meeting regulatory requirements while minimizing manufacturing costs and development timelines (Table 3) (Figure 3).

The QbD approach also requires the identification and control of critical process parameters (CPPs) that directly impact product critical quality attributes (CQAs) [159]. For bacteriophage manufacturing, these parameters include bacterial host strain characteristics and growth conditions, phage propagation parameters such as multiplicity of infection and harvest timing, purification process conditions that affect phage recovery and purity, and formulation parameters that influence product stability and potency. The systematic mapping of relationships between CPPs and CQAs enables manufacturers to establish appropriate process controls and monitoring systems that ensure consistent product quality [8].

### 7.3. Manufacturing Process Development and Challenges

The development of robust manufacturing processes for bacteriophage products presents unique challenges that distinguish these products from conventional pharmaceuticals and even other biological products [8]. The field of phage therapy currently exists in an industrially immature context, with most projects at early clinical trial stages, making it challenging to assemble the multidisciplinary expert teams required for Good Manufacturing Practice (GMP)-compliant manufacturing [8]. The successful implementation of phage manufacturing requires coordination among scientific experts with a deep knowledge of phage biology, technical specialists capable of developing and validating production processes, pharmaceutical experts familiar with regulatory requirements and quality systems, and project management professionals capable of integrating these diverse activities into a coherent development program.

Upstream processing challenges in phage manufacturing center on optimizing bacterial host cultivation and phage propagation systems that can reliably produce high titers of infectious phage particles [31]. The selection and maintenance of appropriate bacterial host strains represent a critical component, as these strains must be genetically stable, capable of supporting robust phage replication, and free from contaminating genetic elements that could compromise product safety. The establishment of bacterial cell banks that can be consistently used for both development and GMP manufacturing activities requires extensive characterization and testing to ensure reproducibility and compliance with regulatory requirements [41].

Downstream processing presents additional complexity due to the need to separate phage particles from bacterial debris, endotoxins, and other contaminants while maintaining phage infectivity and stability [31]. Traditional protein purification methods may not be directly applicable to phage purification due to the larger size and more complex structure of viral particles, necessitating the development of specialized purification protocols. The removal of bacterial endotoxins represents a particular challenge, as conventional endotoxin removal methods may damage phage particles or reduce their biological activity [8]. Recent advances in purification technology, including the use of monolithic chromatographic supports and optimized ultrafiltration systems, have shown promise for addressing these technical challenges while maintaining acceptable process yields.

The complexity of phage cocktail production presents additional manufacturing challenges, as multiple phages must be produced, purified, and formulated together while maintaining the stability and activity of each component [41]. Stability issues under various storage and processing conditions require careful optimization of formulation parameters, including pH, ionic strength, cryoprotectants, and container closure systems. Endotoxin removal becomes particularly complex in cocktail formulations, as different phages may respond differently to purification treatments. The need for standardization across batches necessitates the use of sophisticated analytical methods and quality control systems to ensure consistent product performance.

### 7.4. Quality Control Testing and Analytical Development

The development of appropriate quality control testing methods for bacteriophage products requires consideration of both traditional pharmaceutical testing parameters and phage-specific characteristics that have no direct parallel in conventional drug development [31]. Identity testing for bacteriophage products typically employs molecular methods such as PCR, sequencing, or restriction enzyme analysis to confirm the presence of expected phage genomes and the absence of contaminating phages or bacterial DNA. These methods must be validated to demonstrate specificity, accuracy, precision, and robustness under the conditions of use [8].

Purity assessment represents a critical quality control parameter that encompasses multiple potential contaminants, including host bacterial proteins, endotoxins, nucleic acids from lysed bacteria, and contaminating bacteriophages or bacteria [8]. The development of appropriate analytical methods for these diverse contaminants requires expertise in multiple analytical techniques, including protein analysis, endotoxin testing using Limulus Amebocyte Lysate assays, nucleic acid quantification, and microbiological testing methods. Establishing appropriate acceptance criteria for these tests requires balancing the need for product safety with the practical limitations of purification processes and the intended clinical application.

Potency testing for bacteriophage products presents unique challenges due to the biological nature of the active ingredient and its dependence on interaction with living bacterial targets [78]. Traditional potency assays employ plaque-forming unit enumeration to quantify infectious phage particles; however, these methods may not fully capture the therapeutically relevant activity of the product. Advanced potency testing approaches may include bacterial killing kinetics assays, biofilm disruption assays, or in vivo efficacy models that better reflect the intended clinical application [39]. The development and validation of these more complex potency assays require significant investment in method development and may represent a barrier to entry for smaller biotechnology companies.

Stability testing for bacteriophage products must address both chemical and biological stability parameters over the intended shelf life of the product [8]. Chemical stability refers to the integrity of phage structural proteins and nucleic acids, whereas biological stability focuses on maintaining infectivity and lytic activity over time. The development of appropriate stability testing protocols requires consideration of storage conditions, container closure systems, and the specific stability characteristics of individual phage products. Accelerated stability testing may be employed to support early clinical development; however, long-term, real-time stability data will ultimately be required for regulatory approval.

## 8. Current Clinical Landscape and Trial Outcomes

### 8.1. Global Clinical Trial Portfolio

**Figure 4:**
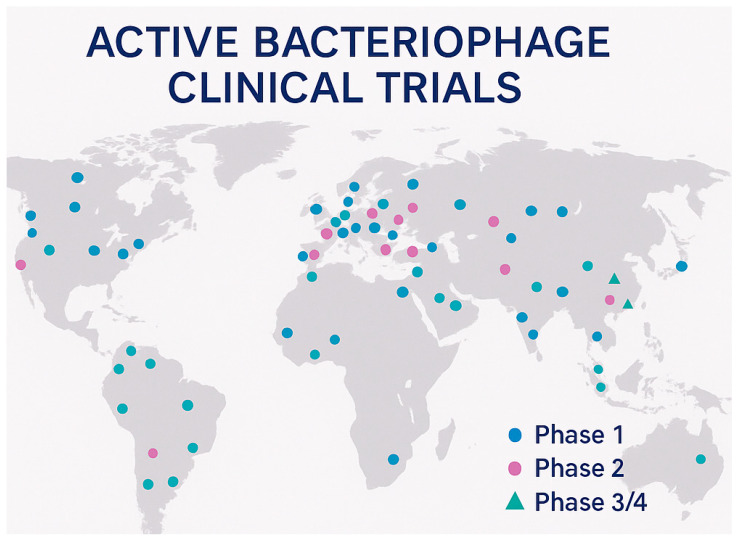
Global distribution of bacteriophage clinical trials.

The contemporary landscape of bacteriophage clinical development encompasses approximately 90 clinical trials worldwide, with 41 studies currently active in the United States, representing a substantial increase in research activity over the past decade [15]. This expansion reflects growing confidence in the therapeutic potential of bacteriophages and increased investment from both public and private sources supporting clinical development [161]. The 2024 revitalization of the bacteriophage field included over $9 million invested in companies BiomX and Locus Bioscience, along with $24 million dedicated by the Biomedical Advanced Research and Development Authority (BARDA) to advance bacteriophage therapies to Phase II trials, demonstrating significant government commitment to this therapeutic approach [162] (Table 4) (Figure 4).

The geographical distribution of clinical trials reflects the global nature of antimicrobial resistance challenges, with studies conducted across North America, Europe, Asia, and Australia [15]. This international scope facilitates the development of diverse phage therapy approaches tailored to regional pathogen epidemiology and regulatory requirements. The variety of clinical indications being investigated ranges from acute infections, such as urinary tract infections and pneumonia, to chronic conditions, including diabetic foot ulcers and prosthetic joint infections, demonstrating the broad therapeutic potential of bacteriophage therapy across multiple medical specialties.

### 8.2. Major Industry-Sponsored Clinical Programs

Armata Pharmaceuticals has emerged as a leading developer of inhaled bacteriophage therapeutics, with multiple clinical programs targeting infections caused by *Pseudomonas aeruginosa* and *Staphylococcus aureus* [145]. The company has completed a Phase 1b/2a clinical trial evaluating its *Pseudomonas* phage cocktail, AP-PA02, in cystic fibrosis patients chronically colonized with *P. aeruginosa*. It is currently conducting a Phase II study in non-cystic fibrosis bronchiectasis patients using the same therapeutic [145]. Additionally, Armata is conducting an ongoing Phase 1b/2a clinical trial to investigate the use of *Staphylococcus aureus* phage in patients with bacteremia, representing one of the first systematic evaluations of intravenous phage therapy for systemic infections.

Locus Biosciences has pioneered the development of genetically engineered bacteriophage therapeutics with its CRISPR-enhanced phage platform [144]. In July 2022, the company launched the ELIMINATE trial, a Phase 2/3 study evaluating the safety, tolerability, pharmacokinetics, and efficacy of LBP-EC01 for treating acute, uncomplicated urinary tract infections caused by multidrug-resistant *E. coli* [144]. LBP-EC01 represents a genetically enhanced cocktail composed of six bacteriophages designed to overcome bacterial resistance mechanisms through multiple targeting strategies. The trial is structured in two parts, with the first part determining optimal dosing for uncomplicated UTIs and the second part conducted as a randomized, controlled, double-blind study. Preliminary results have shown promising outcomes, with urinary *E. coli* levels significantly decreasing within four hours post-administration and complete resolution of UTI symptoms by day ten in all evaluated patients.

TechnoPhage, based in Lisbon, Portugal, is advancing TP-122A, a novel therapeutic comprising three bacteriophages specifically targeting *P. aeruginosa* for the treatment of ventilator-associated pneumonia [163]. The product is administered via nebulization to ensure direct delivery to the lungs, addressing a critical unmet medical need in intensive care settings where *P. aeruginosa* infections are associated with high mortality rates [163]. The Phase 1/2a clinical trial involves 15 adult patients and focuses primarily on assessing safety and tolerability, with secondary endpoints examining the reduction in bacterial load and clinical outcomes.

### 8.3. Academic and Compassionate Use Programs

The Tailored Antibacterials and Innovative Laboratories for Phage (TAILΦR) program at Baylor College of Medicine represents one of the most comprehensive academic phage therapy initiatives, having evaluated 12 cases of customized phage therapy from their production center in 2023 [147]. This program demonstrates the systematic approach required for personalized phage therapy, involving meticulous screening, purification, sequencing, and assessment of phages adhering to stringent pharmaceutical standards. The phages produced by TAILΦR received FDA approval for compassionate use under Investigational New Drug applications, enabling the treatment of patients with severe infections where conventional therapies had failed [147].

The TAILΦR experience provides valuable insights into the practical challenges of implementing personalized phage therapy at scale [148]. Of the 50 requests for phage therapy received by the program, customized phages were successfully produced for only 12 patients, primarily targeting device-related or systemic infections. The detailed analysis of the 38 cases that could not be accommodated reveals the current limitations of personalized phage therapy: patient condition improvement in 8 cases eliminated the need for experimental treatment, treatment delays exceeding 10 weeks occurred in 8 cases due to the complexity of phage isolation and characterization, patient death before treatment initiation occurred in 13 cases, inability to isolate appropriate phages prevented treatment in 5 cases, and unidentified bacterial pathogens precluded targeted therapy development in 4 cases.

The Center for Innovative Phage Applications and Therapeutics (IPATH) at the University of California, San Diego, co-directed by Steffanie Strathdee following her husband’s successful treatment, has been involved in four clinical trials, with additional studies in development [69]. IPATH has established standardized protocols for emergency phage therapy that balance the urgency of clinical needs with regulatory requirements and safety considerations. The program’s experience has contributed significantly to the development of best practices for compassionate use applications and has helped establish clinical protocols that can be scaled to larger patient populations.

### 8.4. International Clinical Experience and Outcomes

A comprehensive retrospective analysis conducted by a Belgian consortium examined the first 100 consecutive cases of individualized phage therapy, spanning 12 countries, 35 hospitals, and 29 cities, from January 2008 to April 2022 [164]. This analysis provided the most extensive evaluation of real-world phage therapy outcomes to date. This multinational study focused on particularly challenging infections, including lower respiratory tract infections, skin and soft tissue infections, and bone infections, representing cases where conventional antimicrobial therapy had failed. The therapeutic approach employed 26 individual bacterial phages and 6 defined bacterial phage cocktails, demonstrating the diversity of phage preparations required for personalized treatment approaches [164].

The clinical outcomes from this comprehensive analysis demonstrate both the potential and limitations of current phage therapy approaches [164]. Treatment yielded clinical improvement in 77.2% of patients and achieved eradication of target bacteria in 61.3% of cases, representing encouraging success rates for a patient population with limited alternative treatment options. However, the analysis revealed a critical finding regarding combination therapy: the likelihood of bacterial eradication was 70% lower when no antibiotics were used concurrently, with an odds ratio of 0.3 and a 95% confidence interval of 0.127–0.749. This finding suggests that optimal clinical outcomes may require combination approaches that leverage the synergistic effects of phages and conventional antibiotics rather than phage monotherapy [165] (Table 5).

These studies also documented important safety and resistance considerations that inform future clinical development [166]. The emergence of bacteriophage resistance was observed in vivo in some patients, confirming that bacterial adaptation to phage therapy occurs in clinical settings as predicted by laboratory studies. However, the synergistic effects of phages and antibiotics observed in vitro were validated in clinical practice, supporting the development of combination therapy protocols that may overcome the individual limitations of each therapeutic modality.

### 8.5. Clinical Studies by Target Bacteria

The distribution of bacteriophage clinical trials by target pathogen reflects the priorities driven by antimicrobial resistance patterns and unmet medical needs (Table 6).

The prevalence of *Pseudomonas aeruginosa* studies reflects the pathogen’s significance in healthcare-associated infections and its extensive mechanisms of antibiotic resistance [1]. *E. coli* studies primarily focus on urinary tract infections, representing a substantial market opportunity with significant unmet medical needs in cases of multidrug resistance. The relatively lower number of studies targeting Gram-positive bacteria may reflect technical challenges in phage isolation and characterization for these organisms.

### 8.6. Regulatory Differences: Systemic vs. Topical Bacteriophage Products

The regulatory pathway for bacteriophage therapeutics varies significantly based on the intended route of administration, with systemic and topical products requiring different developmental approaches and regulatory considerations (Table 7).

### 8.7. Approved Topical Bacteriophage Products

Currently, no topical bacteriophage products have received FDA approval for use in humans as a therapeutic in the United States. However, several products have been approved or are in advanced development, as follows:

#### 8.7.1. Eastern European Approvals

- Pyophage (Eliava Institute, Georgia): Multi-phage cocktail for topical treatment of purulent infections;
- Intestiphage (Eliava Institute): Oral/rectal administration for gastrointestinal infections;
- Sexthaphage (Eliava Institute): Topical treatment for urogenital infections.

#### 8.7.2. Products in Development

- PP1131 (Pherecydes Pharma): Anti-*P. aeruginosa* for burn wounds (Phase I/II completed);
- WPP-201 (Adaptive Phage): Topical treatment for diabetic foot ulcers (Preclinical);
- TP-102 (TechnoPhage): Topical formulation for chronic wounds (Phase I planned).

#### 8.7.3. Regulatory Considerations for Topical Products

Topical bacteriophage products benefit from several regulatory advantages compared to systemic formulations [151]. The limited systemic exposure reduces safety concerns related to immune responses and systemic toxicity, potentially allowing for accelerated development timelines. However, topical products must address unique challenges, including skin penetration, local tolerability, and interaction with wound environments.

The FDA’s guidance for topical antimicrobials provides a framework that can be adapted for bacteriophage products, though specific guidance for topical phage therapy remains under development [151]. Key considerations include demonstrating local efficacy without significant systemic absorption, assessing the potential for skin sensitization, and evaluating product stability under typical storage and use conditions.

### 8.8. Bacteriophage as Food Additives: Regulatory Framework and GRAS Classification

The regulation of bacteriophages as food additives represents a distinct regulatory pathway that has achieved significant success, providing essential precedents for therapeutic applications [142]. The FDA’s Center for Food Safety and Applied Nutrition (CFSAN) oversees bacteriophage products intended for food safety applications under the Federal Food, Drug, and Cosmetic Act.

#### 8.8.1. GRAS (Generally Recognized as Safe) Classification Framework

The GRAS framework provides the primary regulatory pathway for bacteriophage food additives in the United States [141]. Under 21 CFR 170.30, substances can achieve GRAS status through either scientific procedures or through experience based on their everyday use in food before 1958. For bacteriophages, the scientific procedures pathway is typically utilized, requiring a demonstration of safety through published studies and expert consensus (Table 8).

#### 8.8.2. GRAS Notification Process for Bacteriophages

The GRAS notification process for bacteriophage food additives involves several critical components that demonstrate the safety of these additives for their intended use [141]. Manufacturers must provide comprehensive characterization data, including complete genomic sequencing, to confirm the absence of toxin genes, virulence factors, or antibiotic resistance determinants. Manufacturing information must demonstrate consistent production under appropriate quality standards, though full pharmaceutical GMP compliance is not required for food applications.

#### 8.8.3. International Harmonization and Global Approvals

The success of bacteriophage food additives in the United States has facilitated approvals in other jurisdictions through mutual recognition and harmonized standards (Table 9). The European Food Safety Authority (EFSA) has approved several bacteriophage products for food safety applications, though through different regulatory pathways that emphasize novel food assessments rather than additive classifications (Table 9).

### 8.9. Clinical Trial Challenges and Lessons Learned

The PhagoBurn project, developed through collaboration between Pherecydes Pharma (now Phaxiam Therapeutics) and Erytech Pharma, offers valuable insights into the challenges of conducting rigorous clinical trials with bacteriophage therapeutics [153]. This randomized Phase 1/2 study aimed to compare the effectiveness and tolerability of a natural lytic anti-*P. aeruginosa* bacteriophage (PP1131) with standard treatment for wound infections in burn patients across nine burn centers in France and Belgium [153]. The trial design included seven days of daily topical application, followed by a 14-day follow-up period, representing a methodologically sound approach to evaluating the efficacy of topical phage therapy.

The PhagoBurn trial results highlighted critical considerations for phage therapy development that extend beyond simple demonstration of antimicrobial activity [153]. After the complete treatment and follow-up period, investigators observed that PP1131, administered at very low concentrations, reduced bacterial load in burn wounds more slowly than standard antimicrobial treatment. This finding led to the early termination of the trial due to insufficient efficacy compared to the active control, despite the absence of significant safety concerns. The research team concluded that optimizing the PP1131 concentration was necessary, along with further studies, to determine the optimal dosing regimens for clinical efficacy.

The PhagoBurn experience illustrates several important principles for successful clinical development of phage therapy [153]. First, the selection of appropriate phage concentrations requires careful optimization based on preclinical efficacy studies and pharmacokinetic modeling, as insufficient dosing may result in suboptimal clinical outcomes even with otherwise adequate phage preparations. Second, the choice of clinical endpoints and comparator treatments must reflect the intended positioning of phage therapy within the treatment landscape, whether as a first-line therapy, a combination treatment, or a salvage therapy for infections that are resistant to other treatments. Third, the regulatory pathway for phage therapy development benefits from adaptive trial designs that allow for protocol modifications based on interim analysis of safety and efficacy data.

## 9. Strategic Recommendations for FDA Approval

### 9.1. Pre-Clinical Development Strategy

The successful development of bacteriophage therapeutics for FDA approval requires a systematic approach to preclinical development that addresses the unique characteristics of these biological agents while meeting regulatory expectations for safety and efficacy evaluation [151]. Target selection represents a critical first step that should focus on bacterial pathogens with significant unmet medical needs, well-characterized resistance mechanisms, and limited alternative treatment options [1]. Priority targets include multidrug-resistant Gram-negative bacteria such as carbapenem-resistant Enterobacteriaceae, extensively drug-resistant *Pseudomonas aeruginosa*, and *Acinetobacter baumannii*, as well as problematic Gram-positive pathogens including methicillin-resistant *Staphylococcus aureus* and vancomycin-resistant enterococci [4].

The comprehensive characterization of candidate bacteriophages must extend beyond basic host range determination to include complete genomic sequencing and bioinformatics analysis to identify potential safety concerns [68]. Genomic analysis should specifically examine the absence of toxin genes, antibiotic resistance genes, and lysogenic conversion factors that could compromise patient safety or contribute to the development of antimicrobial resistance. Additionally, a phylogenetic analysis comparing candidate phages to known pathogenic or lysogenic phages provides essential safety information for regulatory submissions [92]. The stability of phage preparations under various environmental conditions, including temperature, pH, and ionic strength variations relevant to clinical storage and administration, must be systematically evaluated to support formulation development and establish appropriate handling requirements.

Comprehensive resistance development studies represent a critical component of preclinical development, informing both clinical trial design and post-market surveillance strategies [9,51]. These studies should examine the frequency and mechanisms of bacterial resistance development under controlled laboratory conditions, evaluate the fitness cost associated with phage resistance in target bacterial populations, and assess the potential for cross-resistance between different phages in cocktail formulations. Understanding resistance development patterns enables the design of phage cocktails with complementary resistance profiles, informing clinical monitoring strategies for the early detection of treatment failure.

### 9.2. Clinical Development Pathway Design

Phase I clinical development for bacteriophage therapeutics should prioritize safety and tolerability evaluation while generating preliminary data on pharmacokinetics, pharmacodynamics, and target engagement where technically feasible [151]. The design of Phase I studies must consider the unique characteristics of bacteriophage therapeutics, including their potential for replication at infection sites, their narrow spectrum of antimicrobial activity, and their immunogenicity profile [6]. Safety endpoints should encompass both immediate adverse reactions and delayed immune responses, with particular attention to the development of neutralizing antibodies that could compromise therapeutic efficacy in subsequent treatments.

Dose escalation studies for bacteriophage therapeutics present unique challenges due to the replicating nature of these agents and their dependence on the availability of bacterial targets for therapeutic activity [151]. Traditional maximum tolerated dose determination may not apply to phage therapy, necessitating alternative approaches such as optimal biological dose determination based on target engagement biomarkers or identification of the minimum effective dose through bacterial load reduction measurements. The incorporation of adaptive trial designs allows for protocol modifications based on emerging safety and efficacy data, potentially accelerating development timelines while maintaining patient safety [139].

Phase II clinical development should focus on establishing proof of concept for therapeutic efficacy while continuing to characterize the safety profile in larger patient populations [151]. The selection of appropriate clinical endpoints represents a critical decision that should align with the regulatory guidance for anti-infective drug development while accommodating the unique mechanism of action of bacteriophage therapeutics. Primary endpoints may include reductions in bacterial load, clinical cure rates, or time to clinical improvement, depending on the specific indication and patient population. The incorporation of biomarker endpoints, such as reduction in inflammatory markers or characterization of the immune response, provides valuable mechanistic insights that support regulatory submissions and inform subsequent development phases.

The design of Phase III pivotal trials must address the challenges of conducting large-scale studies with personalized or semi-personalized bacteriophage therapeutics [143]. The use of phage cocktails with broad coverage against target pathogens may enable traditional randomized controlled trial designs. In contrast, personalized phage therapy approaches may require innovative trial designs, such as platform trials or master protocols, that accommodate treatment individualization. The selection of appropriate comparator treatments must consider the current standard of care for target indications while recognizing that many patients eligible for phage therapy may have exhausted conventional treatment options.

### 9.3. Regulatory Engagement and Communication Strategy

Early and frequent communication with the FDA represents a critical success factor for bacteriophage therapeutic development, given the novel nature of these products and the evolving regulatory framework governing their approval [14]. Pre-IND meetings provide an opportunity to discuss development plans, manufacturing approaches, and clinical trial designs with regulatory reviewers before significant resource commitments are made [33]. These meetings should address specific questions regarding the application of existing guidance documents to bacteriophage products, identify areas where additional data generation may be required, and establish a mutual understanding of regulatory expectations throughout the development process.

Type A meetings with the FDA should be reserved for critical safety questions or significant development obstacles that require urgent regulatory input [33]. These meetings are typically granted for serious safety issues, dispute resolution, or situations where development cannot proceed without regulatory clarification. Type B meetings serve as a forum for discussing clinical development plans, encompassing protocol design, endpoint selection, and statistical analysis approaches [33]. These meetings are particularly valuable for bacteriophage development programs due to the limited precedent for these products and the need for regulatory input on novel development approaches.

Type C meetings provide opportunities for technical and regulatory discussions that fall outside the scope of Type A or Type B meetings [33]. These meetings may address manufacturing questions, analytical method development, or comparative effectiveness considerations that inform clinical development strategy. The strategic use of different meeting types throughout development enables developers to maintain an ongoing dialog with regulatory reviewers and address emerging questions before they become significant obstacles to approval.

The preparation of comprehensive briefing packages for FDA meetings requires careful attention to the unique characteristics of bacteriophage therapeutics and the specific questions being addressed [33]. These packages should include relevant preclinical data, manufacturing information, clinical development plans, and particular questions for regulatory discussion. The quality and completeness of briefing packages directly impact the value of regulatory meetings, and the quality of guidance received from the FDA.

### 9.4. Chemistry, Manufacturing, and Controls Strategy

The development of robust Chemistry, Manufacturing, and Controls (CMC) packages for bacteriophage therapeutics requires addressing the unique characteristics of these biological products while meeting established regulatory standards for product quality, safety, and consistency [32,159]. Drug substance development must encompass the complete characterization of bacteriophage active ingredients, including physical and chemical properties, biological activity, and stability characteristics. The establishment of appropriate reference standards for bacteriophage products presents unique challenges due to the biological nature of these agents and the potential for batch-to-batch variation in biological activity.

Manufacturing process development should follow Quality by Design principles from early development stages, with systematic identification and control of critical process parameters that impact product quality attributes [159]. The development of scalable manufacturing processes requires consideration of both upstream bacterial cultivation and phage propagation steps, as well as downstream purification and formulation processes. Process validation studies must demonstrate the consistency and reproducibility of manufacturing processes across multiple batches and varying production scales [8].

Analytical method development for bacteriophage products encompasses traditional pharmaceutical testing parameters along with phage-specific assays that have no direct precedent in conventional drug development [31]. Identity testing methods must be capable of distinguishing target phages from potential contaminants while providing sufficient specificity to meet regulatory acceptance requirements. Potency assays must accurately reflect the therapeutically relevant activity of bacteriophage products, which may require the development of novel biological assays that better predict clinical efficacy than traditional plaque-forming unit enumeration.

Stability testing programs for bacteriophage products must address both chemical and biological stability parameters over the intended shelf life under proposed storage conditions [8]. The development of stability-indicating analytical methods requires consideration of multiple potential degradation pathways, including protein denaturation, nucleic acid degradation, and loss of biological activity. Long-term stability data will ultimately be required for regulatory approval, though accelerated stability testing may support early clinical development activities.

### 9.5. Commercial and Market Access Considerations

The development of successful commercialization strategies for bacteriophage therapeutics requires consideration of multiple factors that distinguish these products from conventional pharmaceuticals [34]. Intellectual property strategy should encompass the composition of matter patents for novel phages, method-of-use patents for specific clinical indications, manufacturing process patents for competitive advantage, and regulatory exclusivities, including orphan drug designation, where applicable. The limited patent life of naturally occurring phages necessitates strategic approaches such as genetically modified phages with enhanced properties or novel formulations that provide proprietary advantages.

Market access preparation should begin during clinical development to ensure a successful product launch following regulatory approval [169]. Health economic modeling demonstrating cost-effectiveness compared to existing treatments provides essential data for payer negotiations and formulary inclusion decisions. The positioning of bacteriophage therapeutics within antimicrobial stewardship programs presents opportunities for differentiated market access, leveraging their narrow spectrum of activity and potential to preserve the effectiveness of antibiotics.

Hospital formulary strategies for acute care settings must address the unique characteristics of bacteriophage therapeutics, including their requirement for pathogen identification, their narrow spectrum of activity, and their potential role in combination therapy protocols [170]. The development of companion diagnostic platforms for rapid bacterial identification and susceptibility testing represents a critical enabler for the successful clinical implementation of bacteriophage therapy.

The establishment of specialized distribution networks may be required for bacteriophage therapeutics, particularly for personalized therapy approaches that require rapid turnaround times between pathogen identification and treatment initiation [69]. Cold chain management and product stability considerations must be incorporated into distribution planning to ensure product quality throughout the supply chain.

### 9.6. Bacteriophage Therapy in Developing Countries and Emerging Markets

The development of bacteriophage therapeutics in developing countries presents unique opportunities and challenges that differ significantly from those encountered in high-income countries [170]. The relatively simple isolation and production processes for bacteriophages, combined with their potential cost-effectiveness, position these treatments as particularly attractive for addressing antimicrobial resistance in resource-limited settings. Lower- and middle-income countries (LMICs), defined as nations with a gross national income per capita of less than USD 4125, bear a disproportionate burden of bacterial infections and antimicrobial resistance [171].

Currently, most publications related to bacteriophage research originate from the U.S. and China [172]. The most productive countries conducting phage therapy research for treating bacterial infections in humans include the U.S., China, Canada, India, Poland, Spain, Australia, and South Korea [172]. However, international cooperation maps have indicated frequent collaboration amongst industrialized nations, with a notable lack of involvement of LMICs. This is especially distressing, considering the incredible opportunity that phage therapy presents to improve global health equity, especially if the production of distinct national or regional phage banks is incentivized [170].

#### 9.6.1. Regional Development Initiatives

Several developing countries have initiated bacteriophage therapy programs tailored to their specific epidemiological needs and bacterial resistance patterns [173,174]. In China, the first documented case of phage therapy dates back to 1958 at Shanghai Jiao Tong University School of Medicine. However, many regulations were not yet established back then, and phage therapy soon lost people’s interest due to the prevalence of antibiotics. Following the first investigator-initiated trial (IIT) conducted by the Shanghai Institute of Phage in 2019, phage therapy rapidly gained momentum [173]. Currently, commercial phage therapy applications must undergo either one of two pathways: fixed-ingredient phage products or personalized phage products, both of which are subject to the restrictive medical technology (IIT) pathway [175].

Currently, only a few companies in China, such as Phagelux, Dalian Hissen, and Shanghai Hi-Tech Bio, are involved in the development of bacteriophage therapy preparations [176]. The global bacteriophage industry is developing steadily, with the worldwide bacteriophage market value reaching 1.1 billion RMB in 2019 and expected to grow to 1.5 billion RMB in 2026, with an annual compound growth rate of 3.8% [177]. The Asia Pacific market is expected to play a more significant role in driving the global market’s development, particularly due to the rapid growth of China, India, and Southeast Asian countries [178].

In India, recognizing the potential of phage therapy, the government is promoting it, with the Indian Council of Medical Research (ICMR) gathering phage researchers and stakeholders to discuss relevant details, paving the way for more specific regulations and research centers in India [174]. The country’s significant agricultural and aquaculture sectors represent critical applications for bacteriophage biocontrol, with several groups working on the isolation and characterization of phages against *Vibrio* species for potential biocontrol applications in shrimp farming [179].

#### 9.6.2. Infrastructure Requirements and Regulatory Capacity Building

The successful implementation of phage therapy in developing countries requires a careful assessment of infrastructure requirements and the local regulatory capacity-building needs [170]. Essential infrastructure includes basic microbiology laboratory capabilities for bacterial isolation and identification, phage production facilities that meet appropriate quality standards, cold chain storage and distribution networks, and healthcare provider training programs for implementing phage therapy.

Regulatory capacity building represents a critical need in many LMICs, where existing frameworks may not adequately address the unique characteristics of bacteriophage therapeutics [157]. International collaboration and technical assistance programs can help establish appropriate regulatory guidelines, provide training for regulatory reviewers, and facilitate harmonization with international standards while accommodating local needs and resource constraints.

#### 9.6.3. Cost Barriers and Public–Private Partnership Opportunities

Cost barriers represent a significant challenge for implementing phage therapy in resource-limited settings, though the relatively simple production requirements for bacteriophages may offer advantages compared to conventional pharmaceuticals [9]. Public–private partnerships offer promising approaches for addressing these challenges, potentially including technology transfer agreements for local production capabilities, tiered pricing strategies that reflect local economic conditions, and collaborative research programs that leverage local bacterial collections and expertise.

International funding mechanisms, including support from organizations such as the World Health Organization, USAID, and private foundations, can play a critical role in facilitating the development and implementation of phage therapy in LMICs [170]. These partnerships could focus on building local capacity for phage research and development, establishing regional phage banks tailored to local resistance patterns, and conducting clinical trials in populations most affected by antimicrobial resistance.

#### 9.6.4. Regulatory Frameworks in Developing Countries

The regulatory landscape for bacteriophage therapy in developing countries varies significantly, with many countries lacking specific guidelines for these novel therapeutic approaches [157]. Compassionate use is a significant pathway that patients rely on to access phage therapy overall, especially in the countries that started to embrace the treatment in recent decades [157]. The representative countries in which compassionate use is a significant access to phage therapy include India, China, and other developing nations where formal clinical trial infrastructure may be limited [166].

The successful implementation of bacteriophage therapy in developing countries requires consideration of multiple factors that distinguish these markets from conventional pharmaceutical development approaches [170]. Unlike traditional drug development that relies on extensive clinical trials and centralized manufacturing, bacteriophage therapy can potentially be developed and implemented through decentralized approaches that leverage local bacterial isolation and characterization capabilities.

The development of appropriate regulatory frameworks for developing countries must strike a balance between the need for patient safety and the practical limitations of resource-limited settings, as well as the urgent medical need for alternative antimicrobial treatments [158]. Regulatory harmonization efforts between developing countries could facilitate the sharing of expertise and resources while reducing the individual burden of establishing comprehensive phage therapy oversight systems.

## 10. Comparative Analysis of Global Regulatory Guidelines

### 10.1. United States FDA Framework

The United States Food and Drug Administration (FDA) regulates bacteriophage therapeutics as biological products under the oversight of the Center for Biologics Evaluation and Research (CBER) [14]. Since 2011, phage therapy has been classified as a medicinal product by the European Medicines Agency. Still, there were disputes about whether it should be classified as a biological medicinal product under Commission Directive 2001/83/EC or an advanced therapy medicinal product, as defined in Commission Directive 2003/63/EC [180]. The FDA framework provides multiple pathways for accessing bacteriophage therapy, including standard Investigational New Drug (IND) applications, expanded access INDs for compassionate use, and single-patient INDs for emergency treatment.

The FDA’s supportive stance toward phage therapy development is evident in statements from industry leaders, who characterize the agency as being “on board” with phage therapy and “very thoughtful and reasonable” in its approach to regulating phage therapeutics [34]. The agency’s 2021 “Science and Regulation of Bacteriophage Therapy” workshop demonstrated collaborative engagement with the phage therapy community, providing developers with direct access to regulatory experts and guidance on navigating the approval process [14].

Key characteristics of the FDA framework include classification as biological products requiring BLA for market approval, standard IND pathway for systematic clinical development, expanded access mechanisms for seriously ill patients, single-patient IND for emergency use, pre-IND meetings and ongoing regulatory dialog, and reasonable Manufacturing Practice requirements aligned with biologics standards [14].

### 10.2. European Medicines Agency (EMA) Framework

Since 2011, phage therapy has been classified as a medicinal product by the European Medicines Agency. Still, there were disputes about whether it should be classified as a biological medicinal product under Commission Directive 2001/83/EC or an advanced therapy medicinal product, as defined in Commission Directive 2003/63/EC [167]. The European regulatory framework for bacteriophage therapy operates within a more complex, multi-jurisdictional system that involves both centralized EMA oversight and national competent authorities.

The EMA stipulated during this workshop that none of the existing regulations adequately fit bacteriophage therapy [158]. Nevertheless, several European countries have found ways to accommodate phage therapy within existing frameworks. Belgium has been particularly innovative in developing pathways for access to phage therapy, implementing a magistral preparation framework that allows for personalized phage therapy under specific conditions [156]. This magistral approach enables hospitals and specialized centers to prepare individualized phage treatments for specific patients under pharmaceutical supervision, providing a practical pathway for personalized medicine approaches that would be difficult to accommodate under traditional industrial pharmaceutical regulations. Poland continues to use phage therapy under Article 35 of the Declaration of Helsinki [181].

The European approach is characterized by classification under Directive 2001/83/EC as biological medicinal products, ongoing development of phage-specific guidance through the European Pharmacopoeia, national variations in implementation and access pathways, complex manufacturing and authorization requirements, and limited precedent for approved phage products [167].

The current provisions that apply to industrial processes of bacteriophage preparations contrast with the theoretical and practical aspects of bacteriophage therapy itself, which is based on a sustainable and tailored patient approach [182]. European regulatory experts have called for adaptive frameworks that differentiate between industrial and personalized approaches while allowing rapid updates of phage preparations [158].

### 10.3. Other Stringent Regulatory Authorities

#### 10.3.1. Health Canada

Health Canada regulates biologics through its Biologics and Genetic Therapies Directorate, following a framework similar to the FDA’s, but with some distinct procedural differences. The Canadian system emphasizes risk-based approaches to drug regulation and has shown openness to innovative regulatory pathways for emerging therapies. Canada’s participation in international harmonization initiatives positions it as potentially receptive to the development of bacteriophage therapy, although specific guidance for phage products has not yet been published.

#### 10.3.2. Japan’s PMDA

Japan’s Pharmaceuticals and Medical Devices Agency (PMDA) operates under the Ministry of Health, Labor, and Welfare (MHLW) and follows ICH-harmonized standards for drug development [183]. Japan’s drug regulation is harmonized with the International Conference on Harmonization (ICH) standards. The notion that Japanese pharmaceutical regulations are significantly different and more stringent than those in the US and Europe is now a dated misconception [183].

Japan has established several innovative programs that could facilitate the development of bacteriophage therapy, including the Sakigake program for breakthrough therapeutics, support for multiregional clinical trials (MRCTs), an emphasis on early development in Japan, and robust consultation and scientific advice programs [184].

The PMDA’s approach to regenerative medical products and gene therapy demonstrates flexibility in adapting regulatory frameworks for novel therapeutic modalities, suggesting potential receptivity to the development of bacteriophage therapy [184]. Regulatory convergence, rather than guideline harmonization, should be promoted due to the variation in those products, and efforts for scientific alignment among international regulatory authorities should be continued [185].

#### 10.3.3. Australia’s TGA

Australia has implemented specific pathways for bacteriophage therapy access through its Therapeutic Goods Administration (TGA) [70,168]. Connected by Phage Australia, a national alliance aiming to standardize phage therapy, researchers and clinicians feature an extensive network across the country based in centers such as hospitals and research institutes [70]. Currently, three pathways allow phage therapy administration: the Special Access Scheme (SAS), Clinical Trial Notification (CTN), and Clinical Trial Exemption (CTX), which require a referral process from a family doctor or specialist to an infectious disease specialist for submission to Phage Australia [168].

### 10.4. Developing Country Regulatory Frameworks

#### 10.4.1. China’s Regulatory Evolution

As early as 1958, the Shanghai Jiao Tong University School of Medicine successfully treated a patient with a *P. aeruginosa* burn infection using phage therapy [186]. However, the ethics approval system was not yet established. It was not until 2019 that the Shanghai Institute of Phage conducted the first in vitro–in vivo (IIT) study of personalized phage therapy at the Shanghai Public Health Clinical Center and Zhongshan Hospital, Fudan University, under the ethics approval framework [186].

China’s approach to regulating bacteriophage therapy has evolved to include fixed-ingredient phage products, following traditional drug pathways, as well as personalized phage products developed through investigator-initiated trials (IITs) [173,175]. Additionally, bacteriophages are classified as restrictive medical technologies for personalized approaches. Additionally, there is growing government support through ICMR initiatives.

#### 10.4.2. India’s Emerging Framework

Recognizing the potential of phage therapy, the Indian government is promoting it, with the Indian Council of Medical Research (ICMR) gathering phage researchers and stakeholders to discuss relevant details [174]. This effort is expected to lead to more specific regulations and the establishment of research centers in India [174]. India’s regulatory development focuses on leveraging the country’s biotechnology capabilities while addressing specific regional resistance patterns and healthcare infrastructure limitations.

### 10.5. Comparative Analysis and Harmonization Opportunities

The comparison of international regulatory frameworks reveals several common themes and divergent approaches, presenting both challenges and opportunities for the development of global bacteriophage therapy [158]. Common challenges include a lack of specific regulatory guidance for bacteriophage products, uncertainty regarding the appropriate product classification, tension between personalized medicine approaches and standardized regulatory requirements, limited precedent for approved products, and complexities in manufacturing and quality control.

Divergent approaches encompass pathway availability and accessibility (compassionate use variations), manufacturing standards and requirements, clinical trial requirements and design flexibility, post-market surveillance and monitoring, as well as participation in international harmonization [158].

Harmonization opportunities include the development of international standards for bacteriophage characterization and quality control, mutual recognition of clinical data and manufacturing standards, coordinated approaches to pharmacovigilance and safety monitoring, shared databases of approved phages and resistance patterns, and collaborative research and development initiatives [187].

The successful global implementation of bacteriophage therapy will require ongoing international dialog and coordination among regulatory authorities to develop appropriate frameworks that strike a balance between innovation and patient safety while addressing the urgent need for alternative antimicrobial treatments [158].

## 11. Future Directions and Emerging Opportunities

### 11.1. Technological Advancements in Phage Engineering

The future development of bacteriophage therapeutics will be significantly enhanced by advances in genetic engineering technologies that enable the rational design of phages with improved therapeutic properties [188]. CRISPR-Cas gene editing systems have revolutionized the ability to precisely modify phage genomes, enabling the development of engineered phages with expanded host ranges, enhanced lytic activity, reduced immunogenicity, and improved stability characteristics. These engineering approaches address many of the traditional limitations of natural phages while maintaining their fundamental mechanism of action and the advantages of specificity.

Synthetic biology approaches offer unprecedented opportunities for designing novel bacteriophage therapeutics with properties not found in natural phage populations [188]. The construction of synthetic phages using modular biological components enables the optimization of individual functional elements such as receptor-binding domains, DNA-replication machinery, and lysis proteins for specific therapeutic applications. These synthetic approaches also facilitate the incorporation of safety features, such as containment mechanisms that prevent uncontrolled phage replication or environmental release.

Protein engineering techniques applied to phage structural components and enzymes provide additional opportunities for therapeutic optimization [18]. The modification of tail fiber proteins to expand the host range or enhance binding affinity enables the development of phages with broader therapeutic coverage or improved efficacy against specific bacterial targets. Similarly, the engineering of lysis proteins such as endolysins can enhance their antimicrobial activity, reduce their immunogenicity, or improve their stability under physiological conditions.

### 11.2. Combination Therapy Strategies

The clinical evidence demonstrating synergistic effects between bacteriophages and conventional antibiotics provides a strong foundation for developing combination therapy approaches that leverage the complementary mechanisms of action of these distinct antimicrobial strategies [38,166]. Rational combination design should consider the specific mechanisms by which phages and antibiotics interact, including phage-mediated sensitization of bacteria to antibiotic action, antibiotic-mediated enhancement of phage penetration into bacterial biofilms, and the potential for combination therapy to suppress the development of resistance to both components.

The development of fixed-dose combination products containing both phages and antibiotics presents opportunities for simplified clinical implementation while ensuring optimal dosing ratios for synergistic activity [165]. These combination products require sophisticated formulation development to maintain the stability and activity of both components throughout the product shelf life, potentially necessitating novel packaging approaches such as dual-chamber systems or lyophilized formulations that are reconstituted immediately before administration.

Sequential therapy protocols represent an alternative approach to combination therapy that may address some of the formulation challenges associated with fixed combinations while maintaining therapeutic benefits [39]. These protocols involve the strategic timing of phage and antibiotic administration to maximize therapeutic efficacy, potentially including phage pretreatment to disrupt bacterial biofilms, followed by antibiotic therapy, or antibiotic therapy to weaken bacterial defenses, followed by phage administration for bacterial elimination.

The integration of bacteriophage therapy with other novel antimicrobial approaches, such as antimicrobial peptides, immunomodulatory agents, or photodynamic therapy, offers additional opportunities for developing comprehensive treatment strategies for multidrug-resistant infections [39]. Natural products such as honey have shown promising synergistic effects with bacteriophages, particularly in topical applications where honey’s antimicrobial and wound-healing properties can complement phage activity against bacterial pathogens. These multi-modal approaches may provide enhanced efficacy while reducing the likelihood of resistance development through multiple simultaneous pressures on bacterial populations.

### 11.3. Personalized Medicine and Diagnostic Integration

The development of rapid diagnostic platforms capable of bacterial identification and phage susceptibility testing within clinically relevant timeframes represents a critical enabler for the implementation of personalized bacteriophage therapy [169]. Point-of-care diagnostic systems based on molecular detection methods, artificial intelligence-enhanced imaging, or biosensor technologies could potentially provide bacterial identification and treatment recommendations within hours rather than days, enabling the timely initiation of targeted phage therapy.

Biomarker-driven patient selection strategies provide opportunities to optimize clinical outcomes by identifying patients most likely to benefit from bacteriophage therapy [6]. The development of predictive biomarkers based on host immune status, bacterial characteristics, or infection site factors could inform treatment selection and enhance treatment success rates. Additionally, pharmacodynamic biomarkers that reflect target engagement and therapeutic response could enable real-time treatment optimization and early detection of treatment failure.

The integration of pharmacogenomic information into bacteriophage therapy protocols represents an emerging opportunity for personalized medicine approaches [6]. Understanding how genetic variations in immune response genes affect patient responses to bacteriophage therapy could inform dosing strategies, treatment duration, and monitoring protocols. Similarly, bacterial genomic information obtained through rapid sequencing approaches could guide phage selection and predict treatment outcomes.

Machine learning and artificial intelligence applications in bacteriophage therapy development offer significant potential for accelerating therapeutic development and optimizing clinical outcomes [93]. These technologies could be applied to phage discovery and characterization, prediction of phage–bacteria interactions, optimization of manufacturing processes, and analysis of clinical outcomes data to identify factors associated with treatment success or failure.

### 11.4. Regulatory Evolution and Global Harmonization

The regulatory landscape for bacteriophage therapeutics continues to evolve as agencies worldwide gain experience with these novel products and develop specialized guidance documents addressing their unique characteristics [187]. The release of further regulatory guidelines by agencies in 2024 demonstrates an ongoing commitment to providing clear development pathways while maintaining appropriate safety standards [187]. Future regulatory developments may include adaptive pathways for accelerated approval based on early efficacy signals, risk-based approaches to manufacturing standards that consider the specific characteristics of different phage products, and platform designations for established phage classes that streamline subsequent product approvals.

International harmonization of bacteriophage therapy regulations presents a critical opportunity to improve global access to these treatments while reducing development costs and complexity [158]. Collaborative efforts between regulatory agencies in different countries could establish mutually acceptable standards for phage therapy development, manufacturing, and clinical evaluation, enabling more efficient global development programs and faster patient access to innovative treatments.

The development of specialized regulatory pathways for emergency use of bacteriophage therapeutics during public health emergencies or for patients with life-threatening infections could provide critical access to these treatments while maintaining appropriate safety oversight [149]. These pathways might include streamlined review processes for emergency use authorizations, expanded access protocols for critically ill patients, or compassionate use frameworks that balance urgent medical needs with regulatory requirements.

Regulatory science initiatives focused on bacteriophage therapeutics could accelerate the development of appropriate testing methods, safety assessment approaches, and efficacy evaluation strategies specifically designed for these unique products [161]. These initiatives might include collaborative research programs between regulatory agencies and academic institutions, the development of reference materials and standards for bacteriophage testing, and the validation of novel analytical methods for product characterization.

## 12. Conclusions

The development of bacteriophage therapeutics represents one of the most promising approaches for addressing the global crisis of antimicrobial resistance, offering unique mechanisms of action that complement and enhance existing antimicrobial strategies [1,4]. The current regulatory environment, while presenting significant challenges, provides clear pathways for the development and approval of these innovative treatments through established frameworks that can accommodate their unique characteristics [5]. The FDA’s demonstrated openness to engaging with phage therapy developers, combined with increasing investment from government agencies and private companies, creates an unprecedented opportunity for bringing these life-saving treatments to patients worldwide [6,161].

The success of bacteriophage therapeutic development requires a comprehensive understanding of multiple interconnected factors that distinguish these products from conventional pharmaceuticals [7]. The biological complexity of bacteriophages necessitates sophisticated approaches to product characterization, manufacturing process development, and quality control that builds upon established precedents from vaccine and biologics development while addressing phage-specific requirements [31,154]. The narrow spectrum of activity, which represents both an advantage and a limitation of phage therapy, requires careful consideration of clinical development strategies, including patient selection, endpoint design, and combination therapy approaches that maximize therapeutic benefit [9,10].

Manufacturing and regulatory compliance represent critical success factors that require early attention and significant investment in specialized expertise and infrastructure [8,41]. The implementation of Good Manufacturing Practice (GMP) standards for bacteriophage products, although challenging, provides a foundation for consistent product quality and regulatory approval. The adoption of Quality by Design principles from early development stages enables the establishment of robust manufacturing processes that can support both clinical development and commercial production while minimizing development risks and costs [159].

The clinical evidence supporting bacteriophage therapy continues to expand through ongoing clinical trials and compassionate use programs that demonstrate both the therapeutic potential and practical challenges of implementing these treatments in clinical practice [13,60,65]. The observation that combination therapy with conventional antibiotics may provide superior outcomes compared to phage monotherapy suggests that optimal clinical application may require integration with existing antimicrobial strategies rather than replacement of traditional treatments [38,166]. This finding has important implications for clinical development strategies, regulatory positioning, and commercial implementation of bacteriophage therapeutics.

The historical context of bacteriophage therapy development, particularly the extensive Soviet experience that maintained institutional knowledge throughout the antibiotic era, provides valuable insights into contemporary development strategies [11,57]. The persistence of phage therapy in Eastern Europe during the Cold War demonstrates both the therapeutic potential of these agents and the importance of sustained institutional commitment to their development [54]. The tragic fate of pioneers like Giorgi Eliava reminds us that scientific progress often occurs despite political and social obstacles and that the dedication of individual researchers can preserve knowledge for future generations [59,60].

The regulatory pathway for bacteriophage therapeutic approval, while complex, provides multiple mechanisms for accessing these treatments based on clinical need and development stage [150]. The availability of expanded access programs for patients with serious or life-threatening infections enables early clinical experience that informs systematic development programs while providing treatment options for patients with limited alternatives [64]. The FDA’s commitment to engaging with developers through pre-IND meetings, guidance document development, and collaborative workshops demonstrates regulatory support for advancing these innovative treatments through appropriate scientific and regulatory processes [14].

A comparative analysis of international regulatory frameworks reveals both opportunities and challenges for the development of global bacteriophage therapy [157,158]. While most regulatory agencies lack specific guidance for these products, the growing recognition of their therapeutic potential has led to increasing regulatory engagement and the development of adaptive pathways for access [156,189]. The success of phage therapy programs in countries like Belgium and Australia demonstrates that existing regulatory frameworks can be adapted to accommodate these innovative treatments while maintaining appropriate safety standards [70].

The emergence of bacteriophage therapy development in countries like China and India represents a significant opportunity for addressing antimicrobial resistance in regions with high disease burdens and limited healthcare resources [173,174]. The relatively simple production requirements for bacteriophages, combined with their potential cost-effectiveness, position these treatments as particularly valuable for improving global health equity [9]. However, the successful implementation of phage therapy in developing countries will require appropriate regulatory frameworks, technical infrastructure, and international collaboration to ensure safety and efficacy standards [69].

Future success in bacteriophage therapeutic development will depend on continued investment in research and development activities that address current limitations while building upon demonstrated strengths [18]. Advances in genetic engineering, synthetic biology, and manufacturing technologies offer opportunities for developing next-generation phage therapeutics with enhanced properties and broader therapeutic applications [188]. The integration of rapid diagnostic technologies, personalized medicine approaches, and artificial intelligence applications could transform bacteriophage therapy from a niche experimental treatment into a mainstream therapeutic option for antimicrobial resistance [169].

The global nature of antimicrobial resistance necessitates international collaboration in bacteriophage therapeutic development, including the harmonization of regulatory standards, the sharing of clinical experience, and the coordination of research efforts [26]. The establishment of international networks for phage therapy development and implementation could accelerate progress while ensuring that benefits reach patients worldwide, particularly in resource-limited settings where antimicrobial resistance poses the greatest threats to public health [67].

As Pierre Kyme from Armata Pharmaceuticals noted, “The FDA is not going to approve phage therapy based on those cases because there’s no way to determine safety. There simply needs to be more clinical trials” [140]. This observation emphasizes that despite encouraging compassionate use experiences and promising preclinical data, systematic clinical development through rigorous clinical trials remains essential for regulatory approval and widespread clinical implementation [49]. The investment of time, resources, and expertise required for this systematic development represents a significant challenge but also an unprecedented opportunity to develop transformative treatments for some of the most challenging infections in modern medicine.

The convergence of urgent medical needs, a supportive regulatory environment, advancing technology, and increasing investment creates favorable conditions for the successful development of bacteriophage therapeutics [6]. For developers entering this field, the combination of these factors provides a clear rationale for investment while highlighting the importance of strategic planning, regulatory engagement, and scientific rigor in development approaches. The potential impact of successful bacteriophage therapy development extends far beyond individual product approvals to encompass fundamental changes in how we approach antimicrobial therapy and resistance management in the 21st century [190].

The future of bacteriophage therapy ultimately depends on the pharmaceutical industry’s ability to navigate complex regulatory pathways while maintaining the highest standards of safety and efficacy [34]. With appropriate planning, execution, and regulatory partnership, bacteriophage therapy can fulfill its promise as a transformative therapeutic approach in the ongoing fight against antimicrobial resistance, providing new hope for patients facing life-threatening infections that no longer respond to conventional treatments [148]. The rich history of phage therapy development, from the pioneering work of d’Herelle and Eliava to the contemporary renaissance driven by clinical necessity, demonstrates that scientific innovation persists through changing political and economic circumstances [11,66]. The current moment represents a unique opportunity to translate nearly a century of accumulated knowledge into approved therapeutics that can address one of the most pressing medical challenges of our time [41].

## Figures and Tables

**Table 1:** Key differences between bacteriophages and conventional antibiotics.

Characteristic	Bacteriophages	Conventional Antibiotics	References
Spectrum of Activity	Narrow (strain/species level)	Broad spectrum (multiple species)	[26,27]
Mechanism of Action	Host-specific lysis and replication	Metabolic inhibition or cell wall disruption	[24,28]
Resistance Development	Co-evolution with host; reversible	Often irreversible, cross-resistance is common	[9,29]
Effect on Microbiome	Minimal impact on non-target bacteria	Significant disruption of commensal flora	[9,30]
Pharmacokinetics	Self-amplifying at the infection site	Traditional dose–response relationship	[6,13]
Manufacturing	Live biological agent; batch variability	Chemical synthesis; consistent batches	[8,31]
Stability	Temperature and pH sensitive	Generally stable under ambient conditions	[8,32]
Regulatory Classification	Biological product (CBER)	Drug product (CDER)	[14,33]
Development Timeline	5–10 years (with regulatory support)	10–15 years (traditional pathway)	[30,34]

**Table 2:** Current FDA regulatory pathways for bacteriophage therapeutics.

Pathway Type	Clinical Scenario	Timeline	Key Requirements	Success Examples	References
Standard IND	Systematic development	5–10 years	Complete preclinical package, GMP manufacturing, controlled trials	Locus Biosciences LBP-EC01, Armata AP-PA02	[143,144,145]
Expanded Access IND	Serious/life-threatening conditions	30–60 days	Treatment IND application, safety data, no comparable therapy	TAILΦR program (12/50 patients treated)	[146,147,148]
Single-Patient IND	Emergency treatment	24–48 h	Emergency IND application, physician justification, informed consent	Tom Patterson case (2016), multiple IPATH cases	[64,69,149]
Emergency Use Authorization	Public health emergency	Days to weeks	EUA request, safety/efficacy data, risk–benefit analysis	Available but not yet utilized for phages	[149,150]

**Table 3:** Critical quality attributes for bacteriophage drug products.

Quality Attribute	Test Method	Acceptance Criteria	Regulatory Rationale	References
Identity	PCR, sequencing, restriction analysis	Match the reference standard	Ensure correct phage product	[68,80]
Phage Titer	Plaque-forming units (PFU)	≥10^8^ PFU/mL (typical)	Therapeutic potency	[77,78]
Purity–Host Proteins	Bradford assay, SDS-PAGE	≤10% total protein	Product quality and safety	[8,31]
Purity–Bacterial DNA	qPCR quantification	≤10 ng/dose	Prevent immune reactions	[8,160]
Endotoxin Level	LAL assay	≤5 EU/kg body weight	Pyrogenicity prevention	[32,159]
Sterility	USP <71> method	No growth	Patient safety	[32,159]
pH	pH meter	6.5–8.0 (typical)	Stability and biocompatibility	[8,31]
Osmolality	Osmometer	280–320 mOsm/kg	Biocompatibility	[32,160]
Particulate Matter	Light obscuration	USP <788> limits	Injectable safety	[32,159]
Container Closure Integrity	Helium leak test	No detectable leaks	Sterility maintenance	[32,159]

**Table 4:** Major active bacteriophage clinical trials (2024–2025).

Company/Institution	Product	Target Pathogen	Indication	Phase	Status	Primary Endpoint	References
Locus Biosciences	LBP-EC01	*E. coli*	Uncomplicated UTI	II/III	Active	Clinical cure rate	[15,144]
Armata Pharmaceuticals	AP-PA02	*P. aeruginosa*	CF lung infection	Ib/IIa	Completed	Safety/tolerability	[15,145]
Armata Pharmaceuticals	AP-PA02	*P. aeruginosa*	Non-CF bronchiectasis	II	Active	Bacterial load reduction	[15,145]
Armata Pharmaceuticals	AP-SA01	*S. aureus*	Bacteremia	Ib/IIa	Active	Safety/PK profile	[15,145]
TechnoPhage	TP-122A	*P. aeruginosa*	Ventilator pneumonia	I/IIa	Active	Safety/tolerability	[15,163]
Adaptive Phage Therapeutics	AdAPT-001	Various MDR	Prosthetic joint infection	I	Planning	Safety assessment	[15]
Pherecydes Pharma	PP1131	*P. aeruginosa*	Burn wound infection	I/II	Terminated	Bacterial load reduction	[153]
TAILΦR/Baylor	Custom cocktails	Patient-specific	Compassionate use	N/A	Ongoing	Clinical improvement	[147,148]

**Table 5:** Clinical outcomes summary from major phage therapy studies.

Study/Program	Patient Population	Treatment Approach	Clinical Improvement	Bacterial Eradication	Key Findings	References
Belgian Consortium (n = 100)	Multi-country severe infections	Individualized phage therapy	77.2%	61.3%	Combination with antibiotics improved outcomes	[164]
TAILΦR Program (n = 12)	Device-related/systemic infections	Customized phage cocktails	75%	58%	38/50 requests could not be accommodated	[147,148]
PhagoBurn Trial (n = 220)	*P. aeruginosa* burn infections	Standardized phage PP1131	No significant benefit	Slower than control	Trial terminated for insufficient efficacy	[153]
IPATH Program (n = 20+)	Compassionate use cases	Personalized treatments	70%	50%	Variable outcomes based on infection type	[69]
Locus ELIMINATE (interim)	*E. coli* UTI	Engineered phage cocktail	100% (small cohort)	85%	Symptoms resolved by day 10	[144]

**Table 6:** Clinical studies by target bacteria (global overview 2020–2025).

Target Bacteria	Completed Studies	Ongoing Studies	Planned Studies	Primary Indications	Key Sponsors	References
*Pseudomonas aeruginosa*	8	12	6	CF lung infections, VAP, burn wounds, chronic wounds	Armata, TechnoPhage, Pherecydes	[15,145,153]
*Escherichia coli*	3	8	4	UTI, bloodstream infections, ESBL infections	Locus Biosciences, BiomX	[15,144]
*Staphylococcus aureus*	4	6	3	Bacteremia, MRSA infections, prosthetic infections	Armata, Adaptive Phage	[15,145]
*Acinetobacter baumannii*	2	4	2	VAP, burn infections, MDR infections	IPATH, TAILΦR	[15,64,147]
*Enterococcus* spp.	1	3	2	VRE infections, UTI	Academic centers	[15]
*Clostridium difficile*	2	2	1	CDI, recurrent CDI	BiomX	[15]
Mixed Gram-negative	5	8	4	Sepsis, wound infections	Multiple	[15]
Total	25	43	22	Various	90 Global Studies	[15,155]

**Table 7:** Regulatory requirements: systemic vs. topical bacteriophage products.

Regulatory Aspect	Systemic Products	Topical Products	References
FDA Classification	Biological product (CBER)	Biological product (CBER)	[14,33]
Phase I Requirements	Full dose escalation, extensive safety monitoring	Limited dose escalation, local tolerability focus	[139,151]
Pharmacokinetics	Comprehensive PK/PD studies required	Limited systemic exposure assessment	[6,151]
Manufacturing Standards	Full GMP compliance from Phase I	GMP compliance with potential relaxed standards	[8,32]
Sterility Requirements	Terminal sterilization or aseptic processing	Bioburden control, antimicrobial effectiveness	[32,159]
Endotoxin Limits	≤5 EU/kg body weight	≤20 EU/g product (topical limit)	[32,160]
Clinical Endpoints	Microbiological and clinical cure	Local bacterial reduction, wound healing	[151,153]
Safety Database	300–600 patients for approval	100–300 patients typically sufficient	[143,151]
Container Closure	Parenteral packaging standards	Topical packaging, stability considerations	[32,159]
Labeling Requirements	Comprehensive systemic safety warnings	Local application warnings, skin sensitivity	[14,151]

**Table 8:** FDA-approved bacteriophage food safety products (2006–2025).

Product Name	Manufacturer	Target Bacteria	Approval Year	Application	GRAS Status	References
ListShield™	Intralytix (Columbia, MD, USA)	*Listeria monocytogenes*	2006	Ready-to-eat foods	GRN 000170	[141,142]
EcoShield™	Intralytix	*E. coli* O157:H7	2007	Ground beef, fresh produce	GRN 000218	[141,142]
SalmoFresh™	Intralytix	*Salmonella* spp.	2009	Poultry, eggs	GRN 000275	[141,142]
ShigaShield™	Intralytix	*Shigella* spp.	2010	Fresh produce	GRN 000320	[141,142]
PhageGuard S	Micreos (Zug, Switzerland)	*Salmonella* spp.	2013	Processed foods	GRN 000435	[141,142]
PhageGuard E	Micreos	*E. coli*	2015	Meat products	GRN 000510	[141,142]
FoodShield™	Intralytix	Multi-pathogen	2018	Various applications	GRN 000745	[141,142]
AquaShield™	Intralytix	Aquaculture pathogens	2020	Fish farming	GRN 000855	[141,142]

**Table 9:** Global regulatory status of bacteriophage food products.

Region/Country	Regulatory Authority	Approval Pathway	Approved Products	Market Status	References
United States	FDA/CFSAN	GRAS notification	8+ products	Commercial	[141,142]
European Union	EFSA	Novel food assessment	3 products	Limited commercial	[158,167]
Canada	Health Canada	Food additive petition	2 products	Commercial	[158]
Australia	FSANZ	Food additive application	1 product	Commercial	[70,168]
New Zealand	FSANZ	Food additive application	1 product	Commercial	[70,168]
Israel	Ministry of Health	Food safety approval	4 products	Commercial	[158]
South Korea	K-FDA	Food additive approval	1 product	Limited	[158]
